# Primary immunodeficiency diseases of adults: a review of pulmonary complication imaging findings

**DOI:** 10.1007/s00330-023-10334-7

**Published:** 2023-11-08

**Authors:** Philippe A. Grenier, Anne Laure Brun, Elisabeth Longchampt, Madeleine Lipski, François Mellot, Emilie Catherinot

**Affiliations:** 1 Department of Clinical Research and Innovation, Hôpital Foch, Suresnes, France; 2 Department of Radiology, Hôpital Foch, Suresnes, France; 3 Department of Pathology, Hôpital Foch, Suresnes, France; 4 Department of Pneumology, Hôpital Foch, Suresnes, France; 5 CEDITH (Centre de Référence Des Déficits Immunitaires Héréditaires), Hôpital Foch Affiliated to Versailles-Saint Quentin University, 40 Rue Worth, 92150 Suresnes, France

**Keywords:** Bronchiectasis, Interstitial lung disease, Pulmonary lymphoma, Pulmonary infection

## Abstract

**Abstract:**

Our objective in this review is to familiarize radiologists with the spectrum of initial and progressive CT manifestations of pulmonary complications observed in adult patients with primary immunodeficiency diseases, including primary antibody deficiency (PAD), hyper-IgE syndrome (HIES), and chronic granulomatous disease (CGD). In patients with PAD, recurrent pulmonary infections may lead to airway remodeling with bronchial wall-thickening, bronchiectasis, mucus-plugging, mosaic perfusion, and expiratory air-trapping. Interstitial lung disease associates pulmonary lymphoid hyperplasia, granulomatous inflammation, and organizing pneumonia and is called granulomatous-lymphocytic interstitial lung disease (GLILD). The CT features of GLILD are solid and semi-solid pulmonary nodules and areas of air space consolidation, reticular opacities, and lymphadenopathy. These features may overlap those of mucosa-associated lymphoid tissue (MALT) lymphoma, justifying biopsies. In patients with HIES, particularly the autosomal dominant type (Job syndrome), recurrent pyogenic infections lead to permanent lung damage. Secondary infections with aspergillus species develop in pre-existing pneumatocele and bronchiectasis areas, leading to chronic airway infection. The complete spectrum of CT pulmonary aspergillosis may be seen including aspergillomas, chronic cavitary pulmonary aspergillosis, allergic bronchopulmonary aspergillosis (ABPA)-like pattern, mixed pattern, and invasive. Patients with CGD present with recurrent bacterial and fungal infections leading to parenchymal scarring, traction bronchiectasis, cicatricial emphysema, airway remodeling, and mosaicism. Invasive aspergillosis, the major cause of mortality, manifests as single or multiple nodules, areas of airspace consolidation that may be complicated by abscess, empyema, or contiguous extension to the pleura or chest wall.

**Clinical relevance statement:**

Awareness of the imaging findings spectrum of pulmonary complications that can occur in adult patients with primary immunodeficiency diseases is important to minimize diagnostic delay and improve patient outcomes.

**Key Points:**

*• Unexplained bronchiectasis, associated or not with CT findings of obliterative bronchiolitis, should evoke a potential diagnosis of primary autoantibody deficiency.*

*• The CT evidence of various patterns of aspergillosis developed in severe bronchiectasis or pneumatocele in a young adult characterizes the pulmonary complications of hyper-IgE syndrome.*

*• In patients with chronic granulomatous disease, invasive aspergillosis is relatively frequent, often asymptomatic, and sometimes mimicking or associated with non-infectious inflammatory pulmonary lesions.*

**Supplementary Information:**

The online version contains supplementary material available at 10.1007/s00330-023-10334-7.

## Introduction

Primary immunodeficiency diseases are a heterogeneous group of inborn errors of immunity exceeding 400 distinct disorders, listed by the International Union of Immunological Societies, mainly defined by specific underlying gene defects [1]. The median age of the first symptoms is 2 years, but 25% of patients develop symptoms after 15 years [2]. Among primary immunodeficiency diseases, primary antibody deficiencies (PADs) are the most common group that have better long-term prognoses and are often diagnosed in adulthood [2, 3]. Furthermore, meticulous medical care and broad-spectrum antimicrobial prophylaxis have enabled patients with other primary immunodeficiency diseases, like those with hyper-IgE syndrome (HIES) or chronic granulomatous disease (CGD) to live to early-to-mid adulthood [4].

Pulmonary complications occurring in patients with primary immunodeficiency disease include infection-related, immune-mediated, and neoplastic diseases. Recurrent acute-onset respiratory tract infections may have long-term effects on the lung architecture, inducing airway remodelling (small airway obstruction and bronchiectasis). The immune dysregulation, characteristic of these diseases can affect multiple organs, including the lungs with non-infectious inflammatory pulmonary infiltration [5]. Finally, some of these diseases are associated with an increased risk of lymphoma, which is a major cause of morbidity and mortality [2, 6].

Computed tomography (CT) has become essential for diagnosing bronchiectasis, small-airway diseases, and non-infectious infiltrative lung disease. CT is also used to monitor disease progression, even if magnetic resonance (MR) imaging offers a radiation-free alternative to CT, particularly for chest surveillance [7]. The aim of this review is to familiarize radiologists with the spectrum of initial and progressive CT manifestations of primary immunodeficiency diseases pulmonary complications, which might achieve earlier diagnosis, thereby potentially improving management. For each of the three entities (PAD, HIES, CGD), we present the imaging features, differential diagnosis, and monitoring of pulmonary complications.

## Primary antibody deficiency

Primary antibody deficiencies (PADs) are the most commonly diagnosed inborn error of immunity, consisting of numerous conditions in which impairment of immunoglobulin production is the predominant pattern. This antibody deficiency may be due to intrinsic B-cell defects, but can also involve functional impairment of other immune cells that promote antibody responses [3]. Tables 1 and 2 summarize the causes, and main clinical, biological, and radiological manifestations of the different disorders considered PADs.

**Table 1:** Pathophysiology and immune characteristics, clinical manifestations, and main chest radiological abnormalities of main primary antibody deficiencies (PADs) [2, 3]

Entity	Pathophysiology and immune characteristics	Clinical manifestations	Main chest radiological abnormalities
CVID with no gene defect specified	Heterogeneous group of disorders involving both B-cell and T-cell immune function Predominant biologic manifestation is hypogammaglobulinemia in individuals aged ≥ 4 years old Normal or decreased number of circulating B cells Low IgG, low IgA and/or IgM Defective antibody responses	Recurrent bacterial infections of the upper and lower respiratory tract Two thirds of patients present noninfectious manifestations including auto-immunity, lymphoproliferative disorders and/or granulomatous infiltration of organs At least 10–20% of CVID patients have ILD Cancer, a major cause of patient deaths, particularly lymphoma and gastric cancers	Airway remodeling: bronchiectasis, bronchial wall thickening, mucoid impactions, atelectasis, mosaic attenuation and expiratory air trapping GLILD: solid and non-solid nodules, areas of consolidation and ground glass opacities often associated with lymphadenopathy. Peri-bronchial and bronchial wall thickening and reticular opacities Pulmonary lymphoma: lymphadenopathy and pulmonary nodules and masses in a peri-lymphatic distribution
IgAD	Characterized by a very low or no circulating IgA with normal IgG and IgM, in individuals aged ≥ 4 years old while IgG subclasses, specific antibodies and circulating B cells are normal	Approximately two-thirds of diagnosed patients are asymptomatic. One third may suffer from bacterial infections, gastrointestinal disorders, autoimmunity and atopy	Bronchiectasis, bronchial wall thickening and mucus plugging Occasionally GLILD: solid and non-solid nodules, areas of consolidation and ground glass opacities, and/or reticular opacities sometimes associated with lymphadenopathy
XLA	Mutations in Bruton tyrosine kinase gene carried on the X chromosome Circulating B cells profoundly decreased Very low IgG and IgA and IgM	Most patients diagnosed before the age of 5 years. Recurrent pneumonias, sinusitis, otitis and conjunctivitis	High rate of bronchiectasis, bronchial wall thickening, mucus-plugging, and atelectasis
IgG-subclass deficiency and SPAD	Characterized by a lack of at least one IgG subclass, frequently associated with IgA deficiency. Normal or immature B cells. Deficiency of IgG1 usually associated with low IgG (CVID or other hypogammaglobulinemia). IgG3 deficiency is the most common in adults. IgG2 deficiency may be associated with poor responses to polysaccharide antigens. SPAD is defined by poor responses to polysaccharide antigens with normal IgG subclasses and normal response to conjugate vaccines	Usually asymptomatic Those with a poor response to polysaccharide antigens have increased risk of infections with capsulated bacteria (invasive and sinopulmonary infections)	Bronchiectasis, bronchial wall thickening and mosaic attenuation and air trapping. Occasionally GLILD particularly when IgA deficiency is associated

Abbreviations:* CVID*, common variable immunodeficiency; *IgAD*, IgA deficiency; *GLILD*, granulomatous lymphocytic interstitial lung disease; *XLA*, X-linked agammaglobulinemia; *SPAD*, specific antibody deficiency

**Table 2:** Pathophysiology and immune characteristics, clinical manifestations, and main chest radiological abnormalities of other primary antibody deficiencies (PADs): combined immune deficiency and syndromes with autoimmunity [2, 3]

Entity	Pathophysiology and immune characteristics	Clinical manifestations	Main chest radiological abnormalities
Hyper-IgM syndrome	Combined immune deficiency characterized by defective B-cell–isotype class-switching. Low IgG and IgA concentrations, and normal-to-high IgM levels The most common and severe form (70%) is X-linked, and caused by CD40-ligand mutations	Sinopulmonary and gastrointestinal infections and auto-immune diseases Enhanced susceptibility to bacterial pathogens and opportunistic infections like *Pneumocystis jiroveci*	Bronchiectasis Hilar lymphadenopathy and perihilar opacities
Activated PI3K-delta syndrome	Combined immune deficiency resulting from autosomal dominant gain of function mutation in PIK3CD Low CD4 T cells. Variable hypogammaglobulinemia, hyper IgM, low serum Ig2 in most patients, poor responses to vaccination	Recurrent sinopulmonary bacterial and Herpes virus infections. Auto-immunity Lymphoid hyperplasia Increased risk of lymphoma Neurodevelomental delay in some patients	Bronchiectasis and airway damage Pulmonary nodules and compressive masses (lymphocytic infiltration)
CTLA-4 haploinsufficiency	Autosomal-dominant immune-dysregulation syndrome with activated T cell compartment Impaired function of T regs. T-cells and B-cells decreased. Variable hypogammaglobulinemia. Regulatory T cells fail to exert their immune regulatory effect, and auto-aggressive immune-cell invasion attacks the lungs	Recurrent infections Lymphocytic organ infiltration, interstitial lung disease. Auto-immune cytopenias	GLILD relatively common: pulmonary solid and non-solid nodules and areas of airspace consolidation
STAT3 gain-of-function mutations	Autosomal dominant gain of function mutation that leads to enhanced STAT3 signaling Increased Th17 cells differentiation. Decreased T regs and impaired function. T-cells and B-cells decreased Variable hypogammaglobulinemia	Multiorgan autoimmunity, infection susceptibility and lymphoproliferative complications Recurrent and severe infections caused by a broad spectrum of pathogens, including opportunistic infections, particularly mycobacterial	Bronchiectasis, bronchial wall thickening, mosaic attenuation and air trapping GLILD: solid and non-solid pulmonary nodules and areas of air space consolidation and/or reticular opacities
LRBA deficiency	Autosomal recessive deficiency that leads to loss of protein expression. LBRA is a cytosolic protein highly expressed in B and T cells and other cellular lineages Low or normal numbers of B cells; Low or normal number of CD4 T cells. T cells dysregulation. Hypogammaglobulinemia	Manifestations typically begin in early childhood with chronic diarrhea (inflammatory bowel disease), auto-immune disorders and organomegaly. Recurrent respiratory infections	Bronchiectasis GLILD: solid and non-solid pulmonary nodules and areas of air space consolidation and/or reticular opacities

Abbreviations:* PI3K-d*, activated phosphoinositide 3-kinase delta; *CTLA-4*, cytotoxic T-lymphocytic associated protein-4; *STAT3*, signal transducer and activation of transcription 3; *LRBA*, lipopolysaccharide-responsive and beige-like anchor protein

Common variable immunodeficiency (CVID) is the most frequently encountered symptomatic PAD. Its estimated prevalence is between 1:25,000 and 1:50,000 affecting men and women equally [3]. CVID is characterized by defective B-lymphocyte differentiation, hypogammaglobulinemia (low IgG and IgA levels), defective antibody responses, and cellular immune defects [8, 9]. It is associated with immune cytopenias, splenomegaly, lymphoproliferative disorders, and granulomatous infiltration of various organs [10].

Selective IgA deficiency (IgAD) is characterized by a very low or no circulating IgA with normal IgG and IgM, and normal IgG subclasses, specific antibodies, and circulating B cells. It is the overall most common PAD, with a prevalence of ~ 1:600 individuals in Europe and North America [3]. Approximately two-thirds of IgAD-diagnosed patients are asymptomatic. One-third may suffer from bacterial infections, gastrointestinal disorders, autoimmunity, and atopy. X-linked agammaglobulinemia (XLA) is due to mutations in the Bruton tyrosine kinase gene carried on the X chromosome and is characterized by a profound decrease of circulating B cells and very low IgA, IgG, and IgM. Most XLA patients are diagnosed before the age of 5 years and suffer from recurrent pneumonia, sinusitis, otitis, and conjunctivitis.

Excepted for IgG-subclass deficiency which presents almost the same prevalence as CVID, the other PAD diseases are rare or very rare (Tables 1 and 2).

### Respiratory infections and airway remodeling

The principal clinical manifestations of PAD are recurrent bacterial infections of the upper and lower respiratory tract. Chronic bronchitis and recurrent pneumonia are common, especially before starting immunoglobulin replacement therapy (IGRT) [3]. Encapsulated bacteria (*Haemophilus influenzae, Streptococcus pneumonia*) are the most causative agents of recurrent infections and can lead to acute complications including pleurisy, empyema, and bronchospasm, and may be of particular importance to the development of chronic lung disease. Furthermore, non-encapsulated strains have also been identified as an important cause of pneumonia and bronchitis in this population. In addition, some patients may develop opportunistic infections due to *Pneumocystis jirovecii* (Figure S1), viruses, tuberculosis (Figure S2), or nontuberculous mycobacteria, typically seen in patients with T-cell deficiencies [5].

**Fig. 1:**
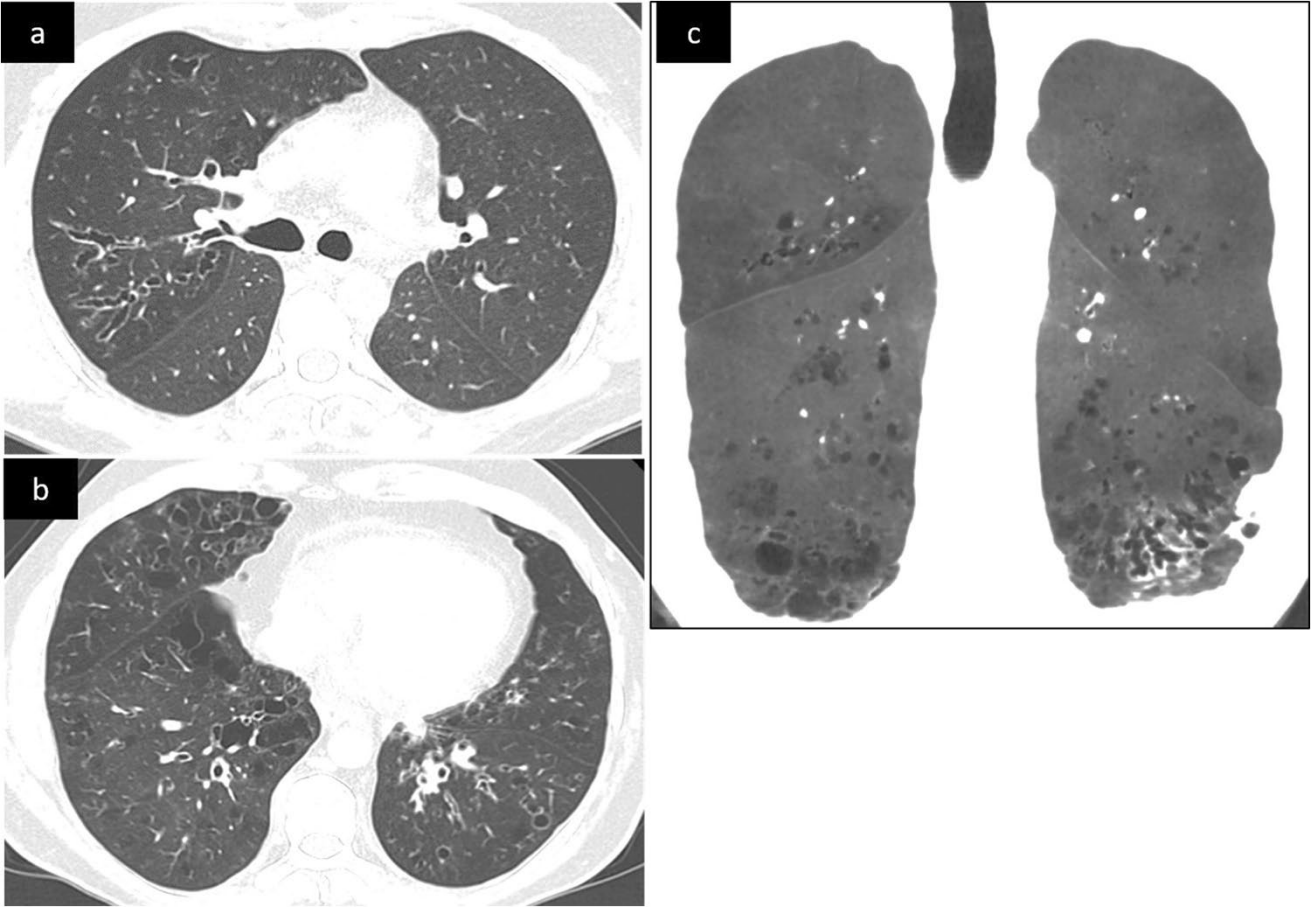
Unenhanced CT scan in a 35-year-old patient with common variable immunodeficiency (CVID), axial (**a**, **b**) and coronal oblique with mIP reconstruction (**c**) images. Diffuse bronchial wall thickening, varicose and cylindrical bronchiectasis with lower and right predominance. The mIP reconstruction shows mosaicism, a few scattered cysts in the lung bases and distal traction bronchiectasis in the left lower lobe

Although early intervention withprophylactic antibiotics and/or IGRT can prevent respiratory infections and pulmonary disease morbidity and mortality, chronic lung disease progresses in many PAD patients. Recurrent infections may instigate a chronic inflammatory response inducing airway hyperreactivity (asthma) and remodeling (bronchial wall-thickening and bronchiectasis), and eventually, irreversible obstruction due to small-airway disease (obliterative bronchiolitis) [8]. The most prevalent PAD-associated radiological manifestation is bronchiectasis associated with small airway disease [9–11] The three morphological bronchiectasis types (cylindrical, varicose, and cystic) reflect increasingly severe bronchial disease and its progression to the more central airway. Bronchiectasis may affect one or more lobes, involving segmental and/or subsegmental bronchi possibly associated with loss of lobe/segment volume (atelectasis) (Fig. 1a, b). Dilated bronchi may contain retained secretions (mucous-plugging, air-fluid levels). Distal mucoid impaction may manifest as parenchymal centrilobular and tree-in-bud nodularity [10]. The other associated findings include bronchial wall-thickening that may precede bronchiectasis onset, areas of lung hypoattenuation, mosaic perfusion pattern (Fig. 1c), and expiratory air-trapping reflecting small-airway obstruction. A multicenter study on the European Chest CT Group cohort included CT data from 232 CVID patients, 28 XLA patients, and 22 patients with other PAD, 80% of patients had radiological evidence of some form of bronchial pathology including bronchiectasis (61%), bronchial wall-thickening (44%), atelectasis (32%) and/or mucus-plugging (29%) [12]. The prevalence of bronchiectasis and mucus plugging, but not of bronchial wall thickening, were higher in the XLA cohort as compared to the CVID cohort (Fig S3). Patients with less severe forms of PAD, such as IgG subclass deficiency, IgAD, and specific antibody deficiency, may also have an increased risk of bronchiectasis (Fig S4) [9].

Although the course of PAD-related bronchiectasis was shown to be similar to bronchiectasis of other causes [13], non-tuberculous mycobacteria or *Pseudomonas aeruginosa* airway colonization can further worsen lung disease in PAD patients with bronchiectasis [3].

### Interstitial lung disease

PAD-related interstitial lung diseases (ILDs) represent a group of diseases relying on a chronic inflammatory and often pro-fibrotic process whose onset is often insidious. An ILD prevalence of at least 10–20% has been reported in CVID, but it is likely underestimated [3]. Occasionally, ILDs may be found in IgAD, particularly when associated with IgG subclass deficiency and with a clinical presentation dominated by autoimmune phenotype [3]. ILD is commonly observed in patients with CTL-4 haploinsufficiency or STAT3 gain-of-function mutations. On the other hand, there is no evidence of ILDs in different cohorts of HIM syndrome and XLA. In terms of severity, there is no data showing specific associations between ILD severity and the type of PAD.

**Fig. 2:**
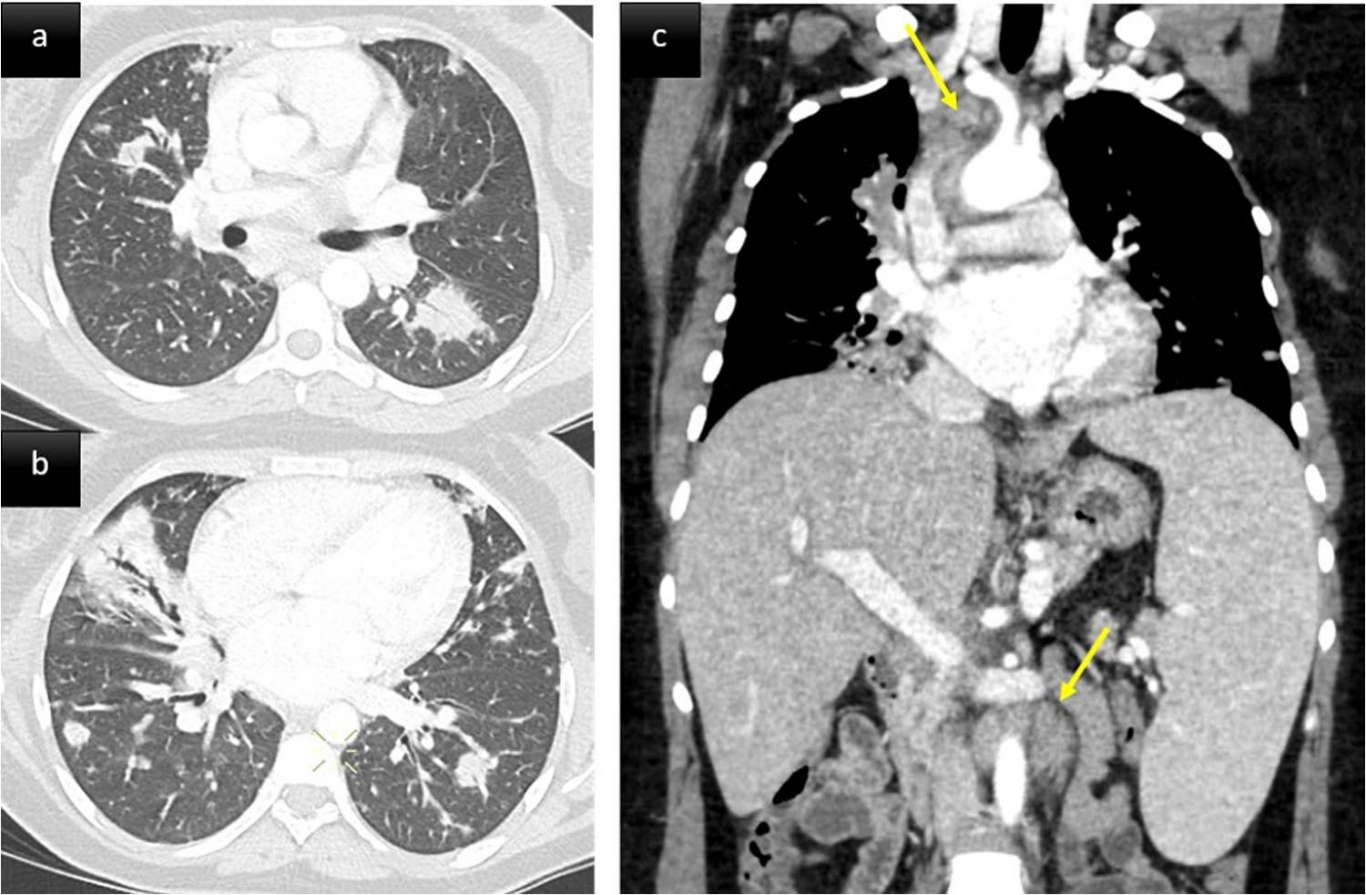
A middle aged woman with CVID. Post contrast thoraco-abdominal CT scan, axial parenchymal (**a**, **b**) and coronal soft tissue (**c**) images. Lower zone predominant broncho centric nodules and airspace consolidation consistent with granulomatous-lymphocytic interstitial lung disease (GLILD). The patient also presented with splenomegaly, mediastinal and retroperitoneal lymphadenopathy (arrows)

**Fig. 3:**
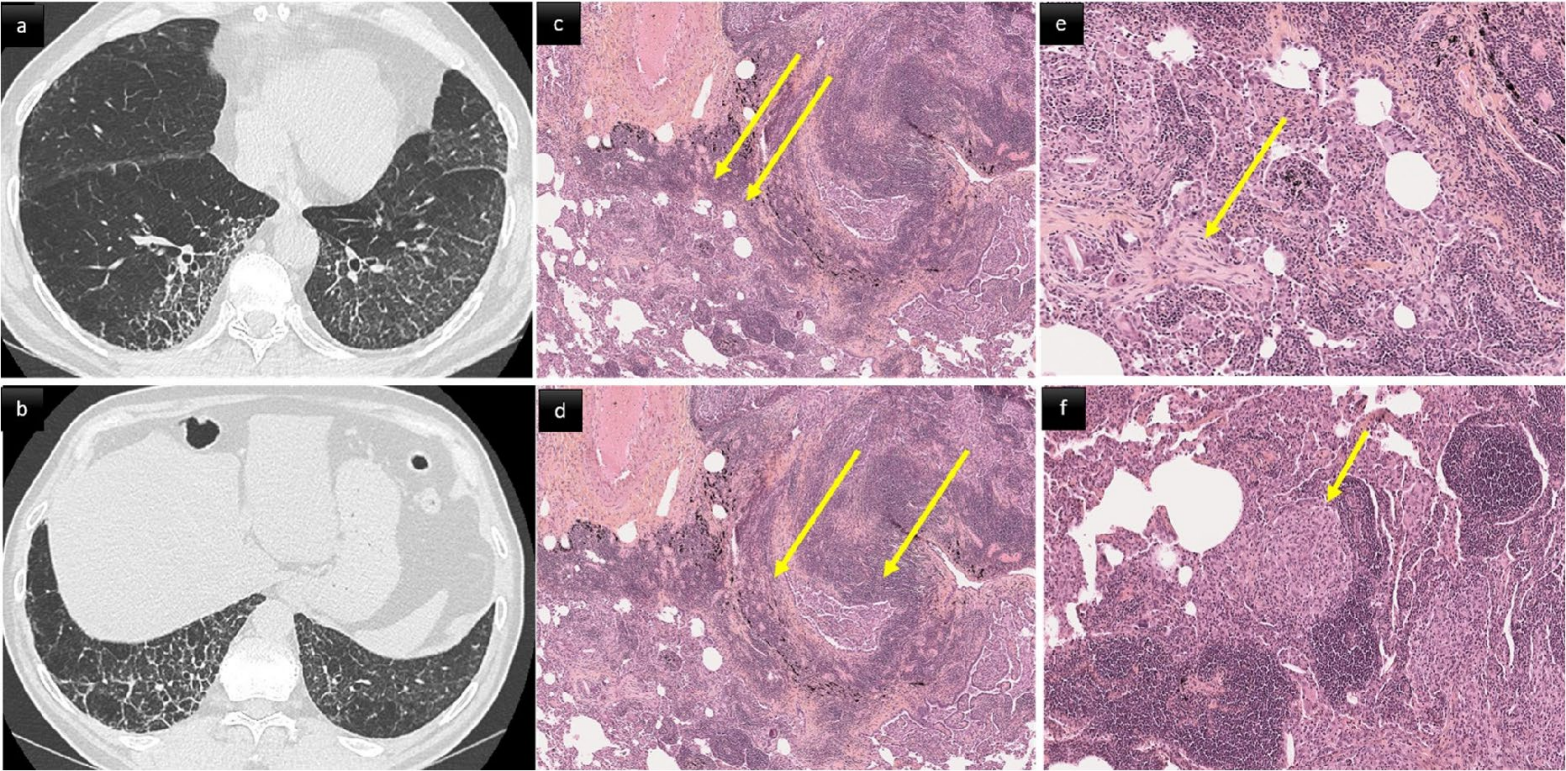
66-year-old male with CVID and biopsy proven GLILD. CT scan (**a**, **b**) and histological (**c**–**f**) correlation. **a**, **b** Smooth and irregular thickening of interlobular septa associated with intralobular reticular lines and architectural distortion in the lower lobes. c: diffuse cellular interstitial infiltrate. The alveolar interstitium is markedly expanded by a dense collection of mature lymphocytes and plasma cells (hematoxylin–eosin, original magnification × 5). **d** Follicular bronchiolitis. Dense airway-centered lymphoid hyperplasia with several small nodular lymphoid aggregates (hematoxylin × 5). **e** Polyps of organizing pneumonia (hematoxylin × 10). **f** Non-necrotizing granulomatous inflammation. Scattered loose aggregates of epithelioid histiocytes often found within interstitial lymphoid background (hematoxylin × 20)

ILD in PAD patients can be considered a pulmonary manifestation of systemic immune dysregulation, representing a serious threat to affected patients’ health. It tends to become symptomatic in later stages and chest CT modifications may foresee clinical and functional manifestations. PAD patients’ ILD characteristics are typically consistent with one or more forms of benign pulmonary lymphoproliferation, frequently associated with granulomatous inflammation and organizing pneumonia. This association led to the umbrella term: granulomatous-lymphocytic interstitial lung disease (GLILD) (Figs. 2 and 3) [14]. GLILD histopathological abnormalities vary and overlap extensively [15] (Fig. 3c–f). Different pulmonary lymphoid-hyperplasia patterns, including follicular bronchiolitis, lymphocytic interstitial pneumonia, lymphocytic infiltrates, and nodular lymphoid hyperplasia, often occur together and are rarely found alone [15] (Fig. 3c-d). The granulomata (Fig. 3f) can vary from poorly-to-well circumscribed, sharing some histological features with sarcoidosis and hypersensitivity pneumonitis. Organizing pneumonia (Fig. 3e) has been reported in many histological specimens [16]. Although a subgroup of patients had interstitial fibrosis with architectural remodeling, only a few had extensive pulmonary fibrosis as the predominant biopsy finding [17].

GLILD diagnosis is based on a combination of radiological and histopathological findings. Typical CT features include large and small solid or semi-solid nodules, areas of consolidation, and ground-glass opacites (GGOs), mainly located in the lower lung zones and having a predominant peri-broncho-vascular and/or subpleural distribution. These lung abnormalities are frequently associated with hilar and/or mediastinal lymphadenopathy [10, 11, 18] (Fig. 2). Thickening of interlobular septa, peri-bronchial and bronchial wall-thickening, reticular opacities, and bronchiectasis may also be seen (Figs. 2 and 3a, b). Lung fibrosis, when present, manifests as lung architecture distortion, traction bronchiectasis, and focal areas of honeycombing. Based on chest CT findings of 51 serial CVID patients, Maglione et al found ≥ 5 pulmonary nodules, bronchiectasis, and GGOs in 34 (67%), 22 (43%), and 18 (37%) patients, respectively [9].

Histopathological confirmation of the diagnosis of GLILD is recommended. Biopsy of the lung tends to be avoided when the radiological findings are highly suggestive or replaced by a liver or lymph node biopsy. 2-[^18^F]-Fluoro-2-deoxy-d-glucose positron-emission tomography combined with CT can visualize active lymphoproliferative sites early during the inflammatory process, showing their systemic distribution [19], potentially identifying those to be more suitable for biopsy than the lung and confirming granulomatous inflammation (Figure S5) [5]. However, in some PAD patients presenting CT features of GLILD, a CT-guided lung biopsy may be necessary to differentiate pulmonary nodules or focal airspace consolidation from malignancies, particularly primary lymphoid lesions.

### Neoplastic diseases involving the lung

Lymphoma is one of the major causes of death in PAD [20]. Primary lymphoid lesions (low-grade B-cell lymphoma, MALT, or Hodgkin diseases) may affect the lungs of these patients. On CT, pulmonary lymphoma is characterized by mediastinal, hilar, and axillary lymphadenopathy with pulmonary nodules and masses in a peri-lymphatic distribution (Figure S6) [11]. These lung opacities may become confluent and contain air bronchograms.

Neoplastic lymphoproliferative diseases, especially mucosa-associated lymphoid tissue (MALT) disease must be considered in the differential diagnosis of GLILD [21]. CT-scan analysis of MALT patients found multi-pattern abnormalities in most, predominantly pulmonary nodules and masses containing air bronchograms of dilated or non-dilated airways [20]. Micronodules, GGOs, and septal lines are less frequent in MALT than in GLILD. MALT diagnosis is easily confirmed by bronchial, transbronchial, CT-guided percutaneous, or surgical lung biopsy (Figure S7) [22].

Lung carcinoma has also been reported in CVID patients, but pulmonary metastases from other cancers appear to be more common than primary lung tumors [23].

### Lung disease monitoring

Even for those without respiratory symptoms, chest CT and pulmonary function tests (PFT) are recommended at PAD diagnosis to look for evidence of chronic lung disease [24]. Then, the next challenge for clinicians and radiologists is the surveillance of these patients. Lung nodule monitoring is entirely relevant, given the heightened malignancy risk of some patients, especially those with CVID. Pertinently, because many of those nodules are benign and wax and wane over time, repeated biopsies might not be necessary. Thus, in the absence of recommended follow-up guidelines, low-dose CT is generally preferred, based on clinical findings with the potential risk of delayed diagnosis for asymptomatic or pauci-symptomatic patients [5]. Contrast is usually not necessary apart from specific indications (haemoptysis, pre-biopsy lymph node assessment, cardiovascular evaluation, suspicion of malignancy). Expiration acquisition is not systematic. Detection of mosaicism and obliterative bronchiolitis can be done by applying the minimum intensity projection technique on a soft kernel window volume and accentuating the contrast (Fig. 1c). Alternatively, MRI provides repeated chest surveillance without radiation exposure and may be useful for clinicians wanting more frequent monitoring (Fig S8) [7]. Actually, one of the main concerns of imaging PAD patients remains the balance between the risks of ionizing radiation and missing bronchiectasis or ILD diagnosis. This is particularly true for CVID patients, who may be more radiosensitive than healthy subjects, as demonstrated after in vitro chromosomal irradiation [25, 26]. Indeed, enhanced radiosensitivity might explain the higher malignancy rate of these patients.

MRI has proved in patients with CVID or IgAD to give similar results as CT for detecting the presence and extent of consolidation, bullae, mucus plugging, bronchial wall thickening, bronchiectasis severity, and nodules [7, 27, 28]. However, CT better identified peripheral airway abnormalities, and MRI performance was weaker at detecting bronchiectasis extension, with a low concordance between MRI and CT in the assessment of the number of bronchial generations. Today, novel MR sequences using so-called ultrashort (UTE) or zero (ZTE) echo-time techniques can provide image quality similar to that of low-dose CT, at a millimeter to submillimeter spatial resolution. Hence, three-dimensional (3D)-UTE MRI allows in vivo assessment of the severity of structural alterations, with the possibility to apply CT imaging scoring systems at a segmental level of precision [29]. Novel respiratory synchronization systems allow for removing motion artifacts, standardizing the lung volume of acquisition without needing an external device, or repeating breath-hold maneuvers. Furthermore, a promising development of functional proton MRI via Fourier decomposition allows the generation of ventilation- and perfusion-weighted maps without any contrast agent. [29]. Evaluation of these new techniques for assessing lung disease in PAD patients is highly expected.

## Hyper-IgE syndrome (HIES)

Hyper-IgE syndrome (HIES) is a group of monogenic primary immunodeficiencies associated with high serum IgE, eczema, and recurrent lung and skin infections. Job syndrome or autosomal dominant HIES is the prototype of these disorders (Table 3). It is caused by dominant negative mutations in STAT3, responsible for a primary immunodeficiency with elevated IgE levels; hypereosinophilia, memory-B-cell lymphopenia, and a low proportion of IL-17-producing TH17 cells. It also affects the connective tissue, skeletal system, vasculature, and dentition [30]. Ninety percent of patients develop recurrent pyogenic caused by *Staphylococcus aureus*, *Sreptococcus pneumoniae*, and *Haemophilus inflenzae* pneumonia, which typically begin in the first several years of life and frequently lead to pulmonary sequelae, such as bronchiectasis and pneumatoceles [11, 30]

**Table 3:** Pathophysiology and immune characteristics, clinical manifestations, and main chest radiological abnormalities of hyper-IgE syndrome (HIES) and chronic granulomatous disease (CGD)

Entity	Pathophysiology and immune characteristics	Clinical manifestations	Main chest radiological abnormalities
Autosomal dominant HIES Also called Job syndrome [24–28]	First cause of HIES, related to loss of function dominant negative *STAT3* mutation Low percentages of IL-17-producing Th17 cells Memory-B-cell lymphopenia Hyper IgE Defective antibody responses	Recurrent pyogenic pneumonia leading to pulmonary sequelae (bronchiectasis and pneumatoceles) complicated by chronic infections due to *Aspergillus fumigatus,* persistent Gram-negative bacilli, like *Pseudomonas,* and non-tuberculous mycobacteria. Atopy Mucocutaneous candidiasis Distinctive facial features (broad nasal bridge), hyperextensible joints, osteoporosis and bone fractures, scoliosis, retention of primary teeth; aneurysm formation	Bronchiectasis. Pneumatoceles Chronic cavitary pulmonary aspergillosis (cavity with thick walls, fungus balls and peri-cavitary infiltrates). Allergic bronchopulmonary aspergillosis (ABPA)-like appearance with bronchiectasis and mucoid impactions
CGD [29–36]	Genetic defect of one of the sub-unit of nicotinamide adenine dinucleotide phosphate (NADPH +) oxidase. This defect renders phagocytic cells unable to produce the reactive oxygen species crucial to killing ingested microorganisms. The most common form (70%) is X-linked, inheritance can also be autosomal-recessive	Recurrent bacterial and fungal infections, mostly affecting the lungs, the liver and the bones Invasive aspergillosis represents the major cause of mortality Chronic non-infectious events may occur in various organs, including the lung	Random pulmonary nodules mainly in the bilateral lower lobes, often associated with GGOs and focal consolidations and masses; cavities and multiple small abscesses may be seen in the focal consolidations and masses. Reticulations, bronchiectasis and pleural effusion can be present. Lymphadenopathies sometimes necrotic

Abbreviations:* HIES*, hyper-IgE syndrome; *CGD*, chronic granulomatous disease; *GGO*, ground glass opacities

**Fig. 4:**
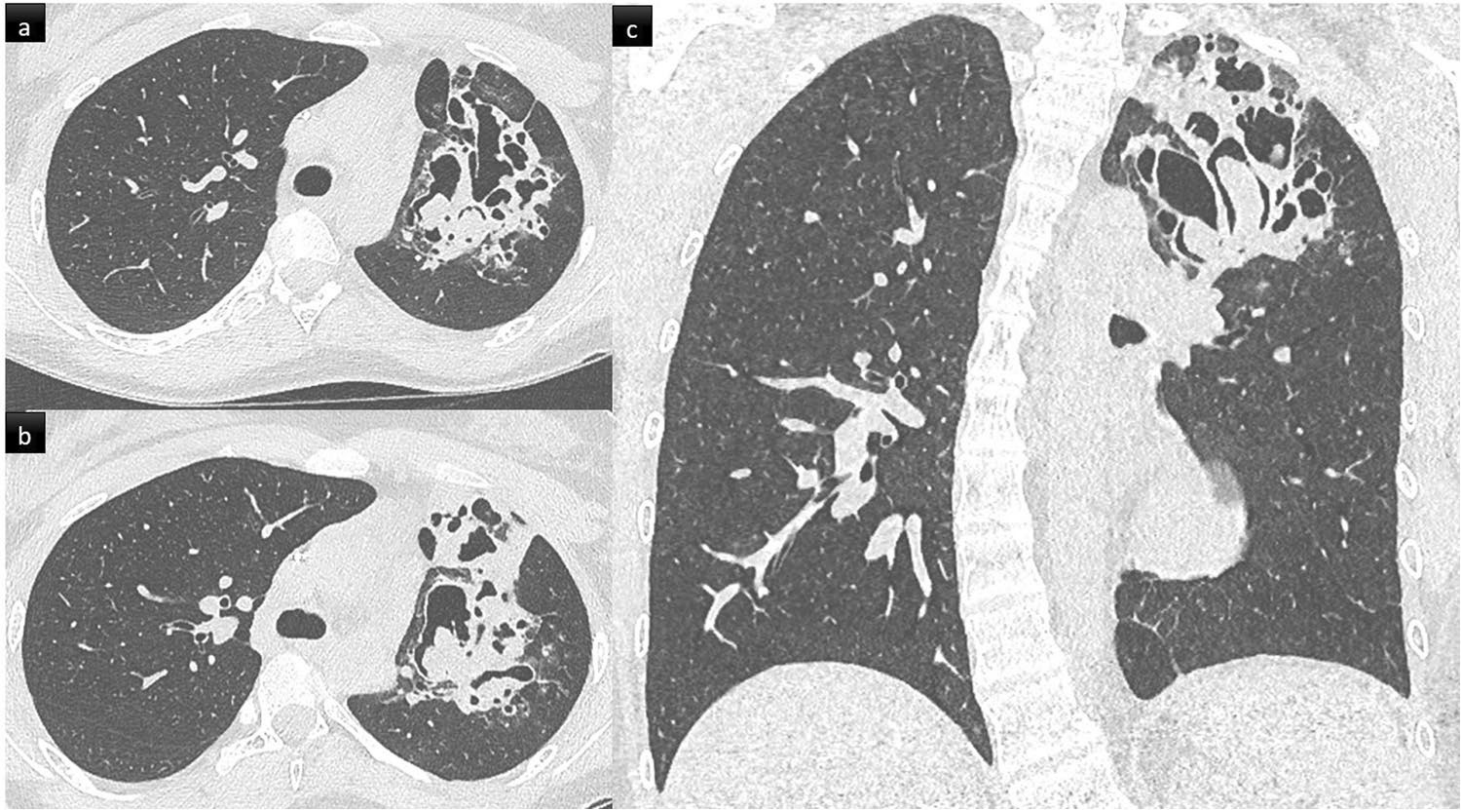
Chronic aspergillosis in a 32-year-old patient with autosomal dominant hyper IgE syndrome (HIES). Axial (**a**, **b**) and coronal mIP (**c**) CT scan images. Large cavities in the left upper lobe (**a**, **c**) with evidence of intra cavitary aspergillomas. Note the thoracic scoliosis, which is a frequent finding in HIES

Once the lung parenchyma is damaged by pyogenic pneumonia, the list of infecting agents expands to include *Aspergillus fumigatus,* persistent Gram-negative bacilli, like *Pseudomonas,* and non-tuberculous mycobacteria [30]*.* Commonly, secondary infections with aspergillus species and/or Gram-negative infections cause clinically significant haemoptysis and/or become increasingly resistant to antimicrobial agents. Most aspergillosis-related infections develop in pre-existing pneumatocele and bronchiectasis areas, leading to chronic airway infection [31] (Fig. 4). The CT pulmonary aspergillosis spectrum described in autosomal dominant HIES include (1) aspergillomas with fungus balls within pre-existing lung cavities (bronchiectasis or pneumatoceles), (2) chronic cavitary pulmonary aspergillosis with cavity thickness, fungus balls and peri-cavitary infiltrates, and (3) allergic bronchopulmonary aspergillosis (ABPA)-like with bronchiectasis and mucoid impactions taking the appearance of toothpaste-shaped/finger-in-glove opacities (Figure S9) [25]. Mixed patterns and episodes of invasive aspergillosis can also occur [32]. The CT evidence of various patterns of aspergillosis developed in severe bronchiectasis or pneumatocele in a young adult should raise the potential diagnosis of autosomal dominant HIES.

### Monitoring lung disease

Prophylactic antimicrobials to prevent pyogenic pneumonia and early recognition and treatment of infection are essential to try and prevent the development of pneumatoceles and bronchiectasis. When chronic fungal infection of pneumatoceles or bronchiectatic airways is present, long-term administration of a mold-active antifungal seems to minimize the spread of infection and associated bleeding. Because antifungals cannot always effectively treat aspergillomas forming inside pneumatoceles, resection has been performed in some patients. However, less than 50% of these lung surgeries may lead to complications with prolonged bronchopleural fistulae, often persisting for months and causing empyema and sometimes requiring re-intervention [33]. Haemoptysis can be a major cause of morbidity and mortality, likely from abnormal vasculature associated with the chronic infection (Fig S10) [34]; sometimes arterial embolization or surgical resection may be required. Invasive fungal disease typically occurs in the fourth decade of life and only when there are concurrent anatomical lung defects from previous bacterial pneumonias. Despite therapy, mortality from invasive fungal disease is significant. Finally, several successful hematopoietic stem cell transplants (HSCT) in patients with autosomal dominant HIES have been published, prompting increased consideration of this option [30].

## Chronic granulomatous disease

Chronic granulomatous disease (CGD) is a rare inherited PID caused by a genetic defect of the nicotinamide adenine dinucleotide phosphate oxidase. The mode of inheritance can be X-linked or of autosomal recessive form (Table 3). This defect renders phagocytic cells unable to produce reactive oxygen species (ROS) which are crucial to killing ingested microorganisms [35]. CGD is estimated to occur in ~ 1/200,000–250,000 live births in the USA [36]. The disease is characterized by recurrent life-threatening infections and inflammatory manifestations [36]. The lung is the main affected organ. Pulmonary infections are often severe and invasive aspergillosis represents the major cause of mortality. Chronic non-infectious events may occur in various organs including the lung. Anti-microbial prophylaxis with itraconazole and sulphamethoxazole and improved management of infectious and inflammatory complications have, however, dramatically improved CGD outcomes over the last decades [37]. Patients now easily reach adulthood [38].

### Respiratory infections

**Fig. 5:**
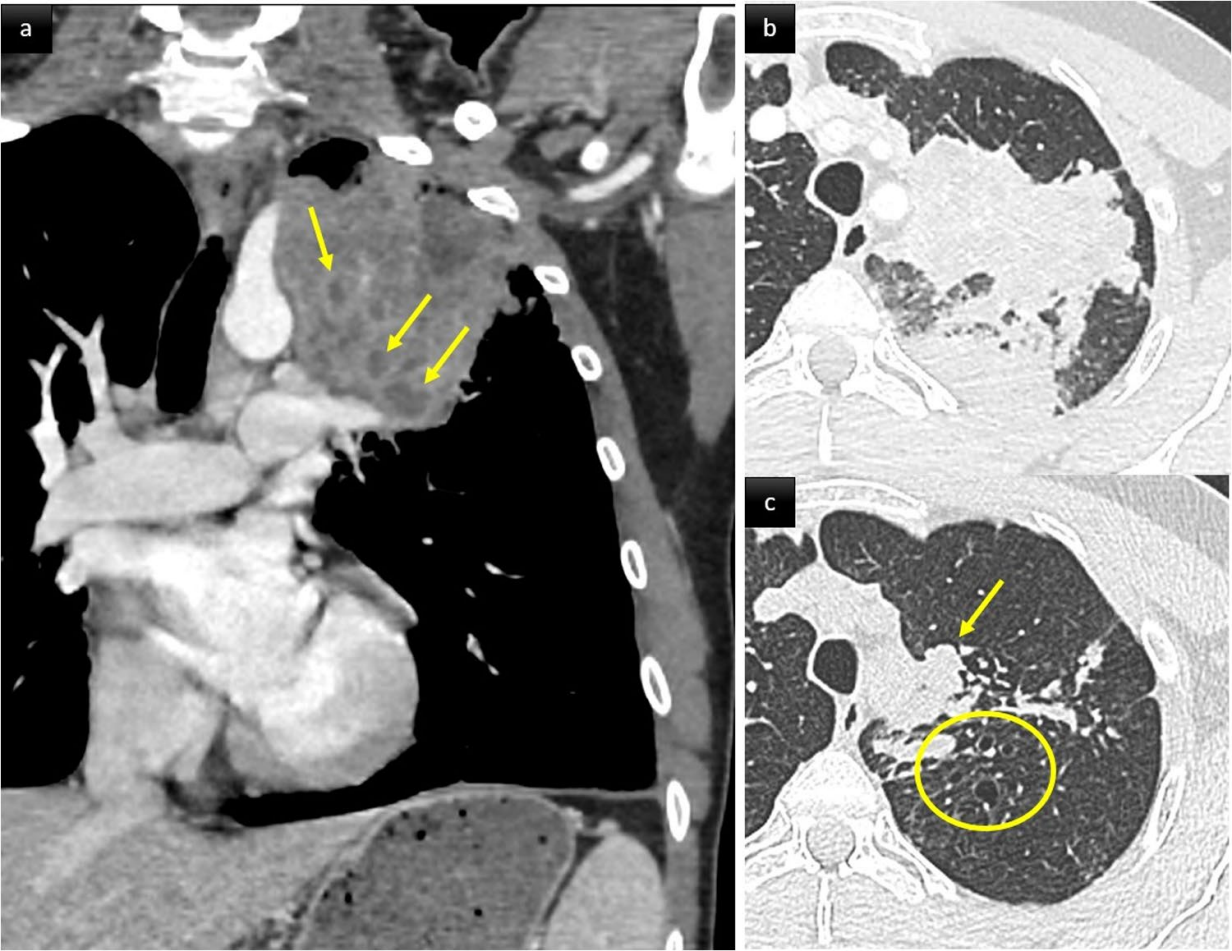
Chronic granulomatous disease (CGD) revealed by a mixed pattern of aspergillosis in a 41 yo man. Coronal post contrast (**a**) and axial parenchymal (**b**, **c**) CT images. Large partially necrotic consolidation in the left upper lobe. Tubular opacities less dense than airspace consolidation are consistent with plugged dilated airways (arrows). Note the presence of underlying varicose bronchiectasis in the same lobe (circle)

The various pathogens identified in the case of respiratory infections include bacteria, fungi, and *Mycoplasma* [39]. Furthermore, mycobacterial disease is also relatively common in patients with CGD living in countries in which tuberculosis is endemic, the BCG vaccine is mandatory [40]. At diagnosis, chest CT shows random pulmonary nodules ranging from 1 to 3 cm in diameter and mainly in the bilateral lower lobes often associated with GGOs and focal consolidations and masses [39, 40]. Cavities and multiple small abscesses may be seen in the focal consolidations and masses; some patients also have reticulations, bronchiectasis, and pleural effusion(s) (Fig. 5). Fungal infection features tend to be unusual. Numerous patients have neither fever nor respiratory symptoms [39]. Microbiological identification, mostly *Aspergillus fumigatus* and *Aspergillus nidulans*, is obtained on bronchoalveolar lavage or lung biopsy analysis. *A. nidulans* is clearly over-represented in CGD, compared with other immunodeficiencies, suggesting host–pathogen interactions and the role of NADPH + oxidase within *Aspergillus* species [41]. Single or multiple, uni- or bilateral pulmonary nodules (< 30 mm), with or without a halo sign, might represent the first radiological findings of pulmonary fungal infection in CGD [42]. In the analysis of CT scans of 23 adult CGD patients diagnosed with fungal infections, Salvator et al found airspace consolidation, nodules, reticulation, and GGOs in 20, 12, 10, and 11 patients, respectively (FIG S11); chest wall invasion was detected in six patients, with rib lysis in three, subcutaneous abscesses in two, and subclavian artery compression in one (Figure S12) [39]. Lymphadenopathies can be present, sometimes necrotic [11].

### Noninfectious pulmonary events

**Fig. 6:**
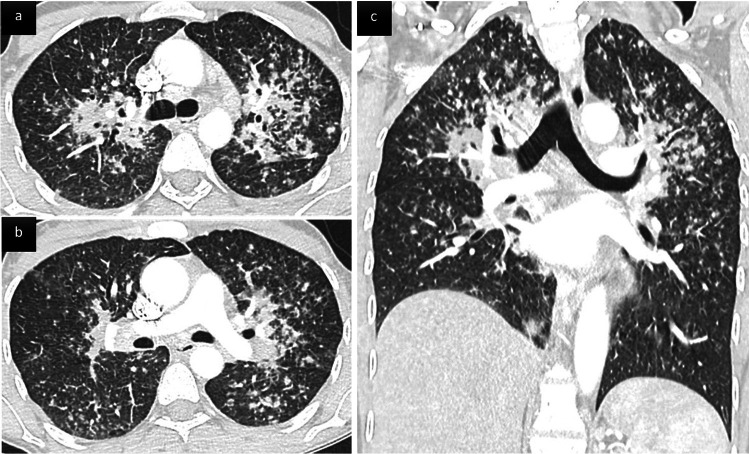
Sarcoid-like infiltrative lung disease following systemic aspergillosis in a 46 yo patient with chronic granulomatous disease (CGD). Axial (**a**, **b**) and coronal (**c**) CT scan images show innumerable small nodules with a peri-lymphatic distribution (peri-broncho-vascular, centrilobular and subpleural) predominant in the upper parts of the lungs, associated with perihilar confluence of nodules, and enlarged hilar and mediastinal lymph nodes. Granulomas were confirmed on transbronchial biopsies

Non-infectious inflammatory pulmonary manifestations have been reported in 23–28% of CGD patients [39, 43]. They were more frequent in the X-linked inheritance group. Two different CT patterns have been described. The first is made up of circumscribed nodules or airspace consolidation, sometimes arising after a respiratory infection; lung biopsy histology may find granuloma and neutrophilic or eosinophilic micro-abscesses. The second is made up of variable infiltrative lung patterns including disseminated micronodules and peri-bronchial thickening (sarcoid-like pattern) (Fig. 6) or non-specific interstitial pneumonia with GGOs, reticulations, and septal-thickening [39]. Given that infection and chronic inflammation are clearly linked in CGD, a high percentage of those inflammatory events occur simultaneously with an infection, mainly aspergillosis, frequently making lung biopsy necessary to differentiate them from uncontrolled infection, particularly for circumscribed nodules or airspace consolidation.

### Monitoring lung disease

Protocols of continuing intensive surveillance and monitoring of compliance with anti-infective regimens may significantly improve the quality of life and long-term survival in patients with CGD [36]. Infections with Aspergillus species have become the major cause of infectious complications and death in patients with CGD. Prophylactic and therapeutic measures are needed to further increase life expectancy and quality for patients with CGD [38].

Pulmonary complications indicate poor prognosis and are frequently responsible for sequelae. CT images of retractile lung scarring have been reported in a series of adult CGD patients. Salvator et al examined nine chest CT scans after treatment completion; all showed radiological scarring with local fibrosis, septal-thickening, bronchiectasis, and paraseptal emphysema [39]. The burden of these complications raises the question of respiratory monitoring of CGD patients. Systematic low-dose CT every two years has been recommended [39]. For any new respiratory event, management must be initiated early, including CT scans and invasive diagnostic techniques, even lung biopsy.

## Conclusions

With improvements in diagnosis and therapy, an increasing number of patients with primary immunodeficiency diseases now survive into adulthood. Hence, radiologists should familiarize themselves with imaging findings associated with these diseases, to raise the diagnosis of primary immunodeficiency disease when confronted with suggestive features or associations and detect complications in the specific subgroups.

### Supplementary Information

Below is the link to the electronic supplementary material.

Supplementary file1 (PDF 1677 KB)

## Abbreviations

ABPA: Allergic bronchopulmonary aspergillosis
CGD: Chronic granulomatous disease
CT: Computed tomography
CVID: Common variable immunodeficiency
GGO: Ground-glass opacity
GLILD: Granulomatous lymphocytic interstitial lung disease
HIES: Hyper-IgE syndrome
IgAD: Selective IgA deficiency
IGRT: Immunoglobin replacement therapy
ILD: Interstitial lung disease
MALT: Mucosa-associated lymphoid tissue
MR: Magnetic resonance
PAD: Primary antibody deficiency
PFT: Pulmonary function tests
XLA: X-linked agammaglobulinemia

## Declarations

### Guarantor

The scientific guarantor of this publication is Phillipe A Grenier.

### Statistics and biometry

No complex statistical methods were necessary for this paper.

### Study subjects or cohorts overlap

No study subjects or cohorts have been previously reported.

### Methodology

• performed at one institution
