# Supplementary material for: Primary immunodeficiency diseases of adults: a review of pulmonary complication imaging findings
Source: Eur Radiol. 2023 Nov 8;34(6):4142–54. doi: 10.1007/s00330-023-10334-7 (PMC11166740; doi:10.1007/s00330-023-10334-7)
Supplement: Supplementary file 1 — Supplementary file1 (PDF 1677 KB) [file 330_2023_10334_MOESM1_ESM.pdf]

Supplementary material

Figure S1-jpeg

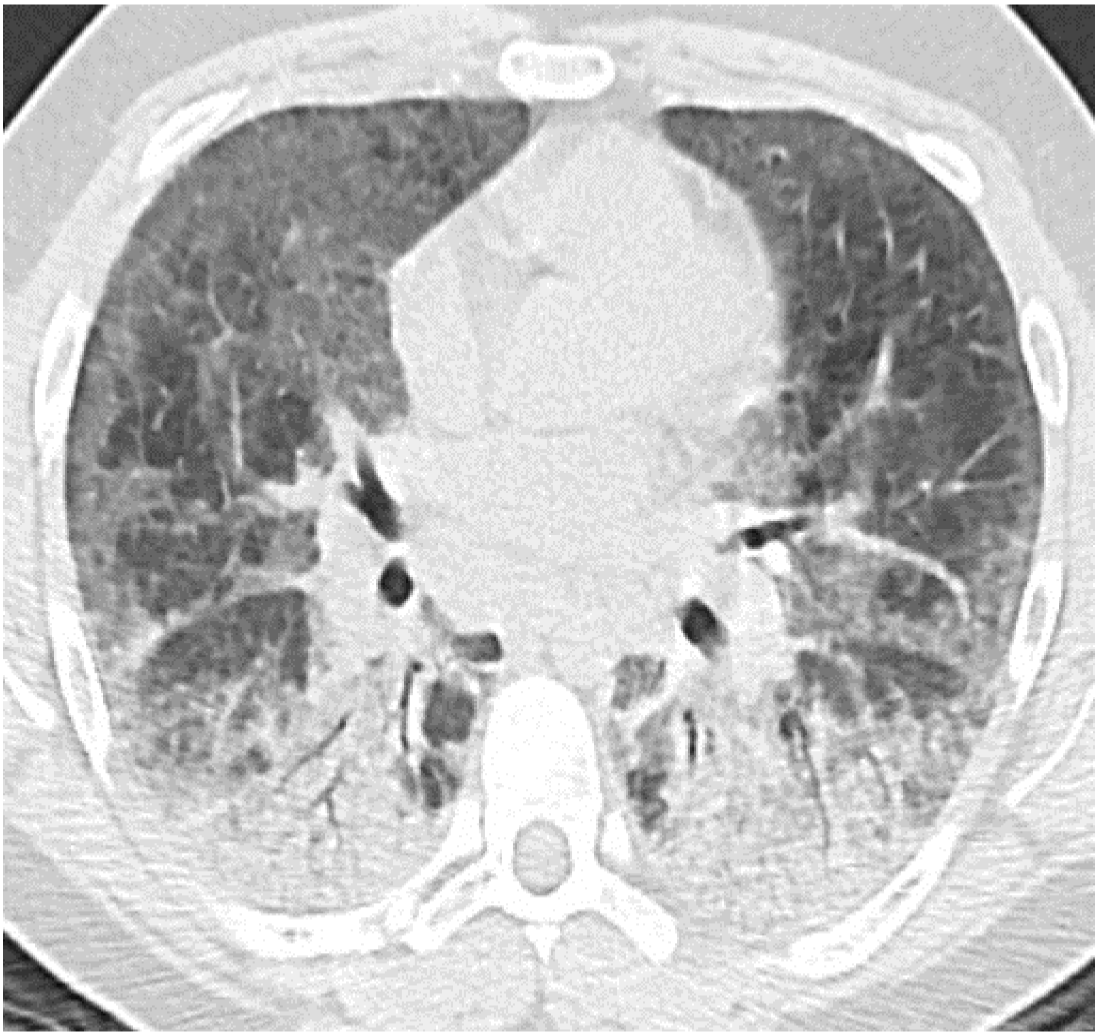

Figure S1 Common variable immunodeficiency in a 17 yo patient revealed by *P. jirovecii* infection.

Figure S2-abc_jpeg

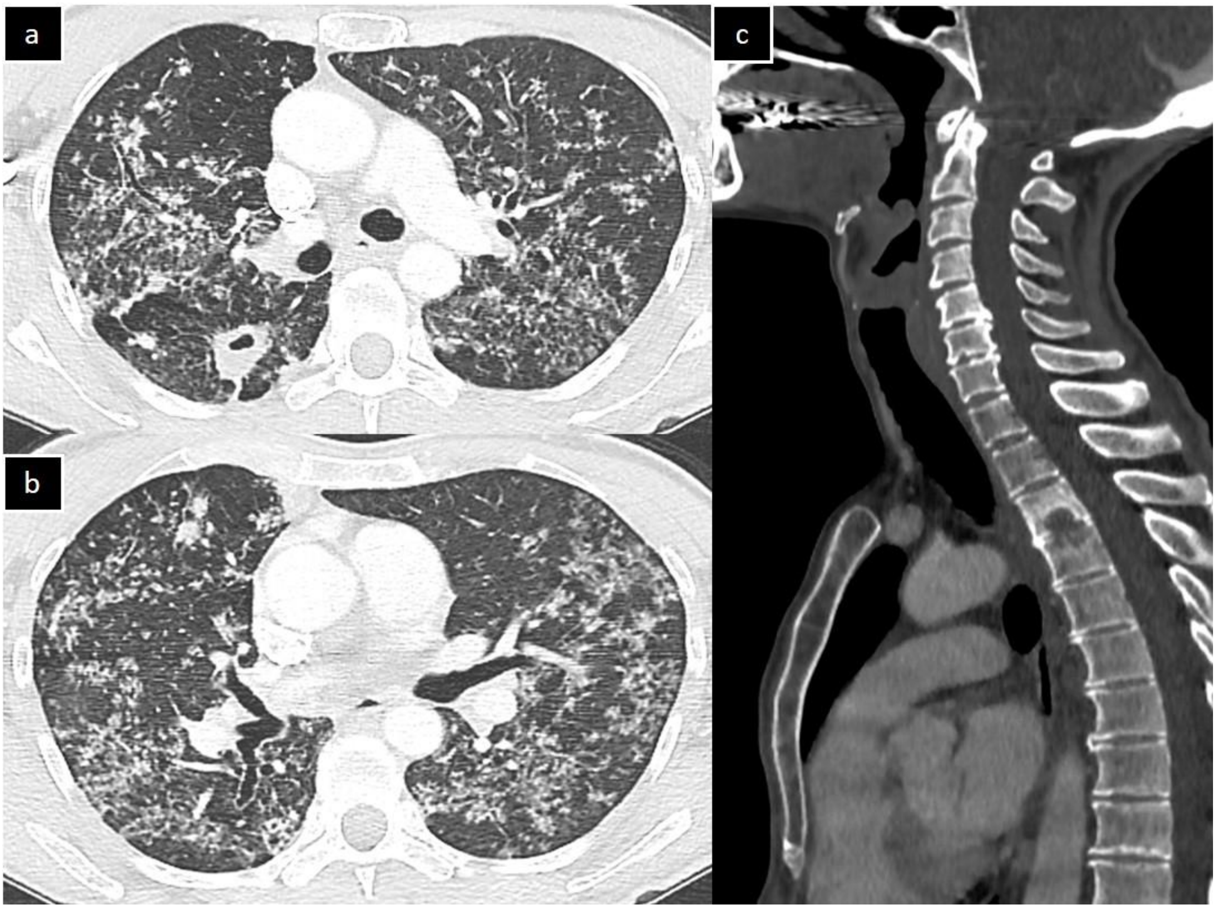

Figure S2- Disseminated tuberculosis in a 52 yo woman with CVID. Axial Ct images (a, b) show innumerable centrilobular nodules and a cavitating lesion in the right lower lobe. There is evidence of T3-T4 spondylitis on the sagittal view (c).

Figure S3_jpeg

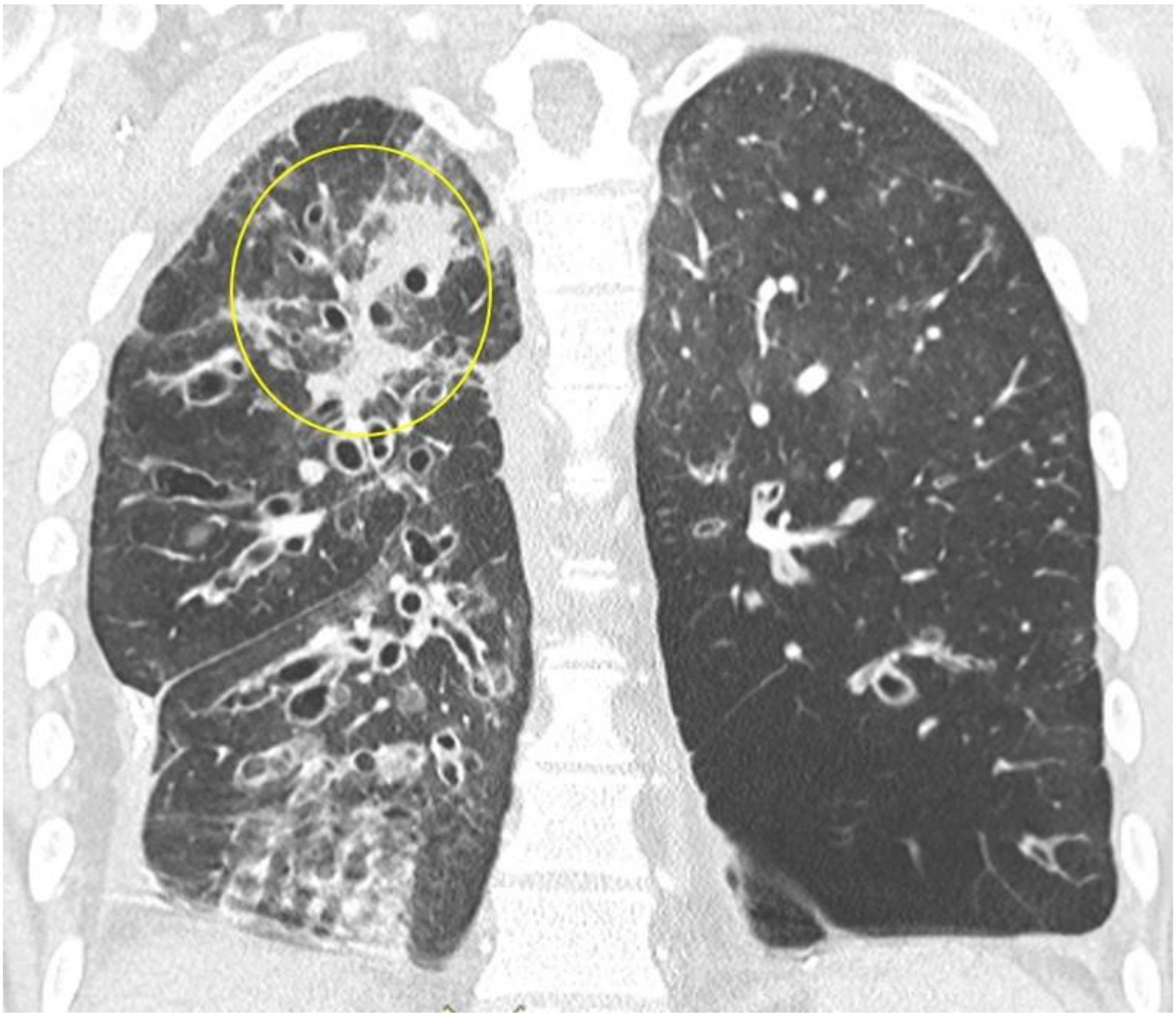

Figure S3- 35 yo patient with X-linked agammaglobulinemia (XLA)), who presented at ICU for fever and worsening dyspnea. The CT scan shows extensive varicose bronchiectasis in the right lung and left lower lobe, associated with evidence of constrictive bronchiolitis in the left lower lobe. Diffuse bronchial wall thickening, and superimposed RUL opacities (circle) related to acute *H influenza* infection

Figure S4_jpeg

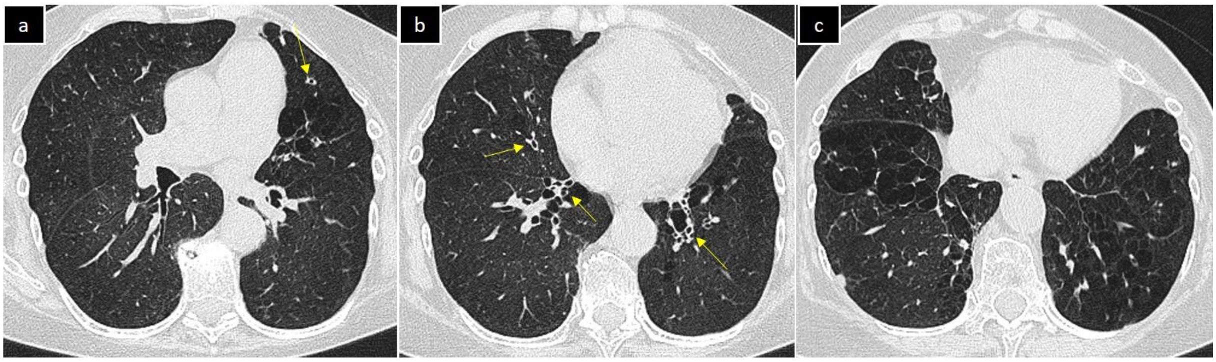

Figure S4; Axial CT images in a 73 patient with selective IgA deficiency, asymptomatic. There are mild cylindrical bronchiectasis in the lower lobes and left upper lobe (arrows), associated with bronchial wall thickening. Peribronchovascular areas of hypoattenuation in the same areas, probably related to paracicatricial emphysema and/or obliterative bronchiolitis

Figure S5-abcd_jpeg

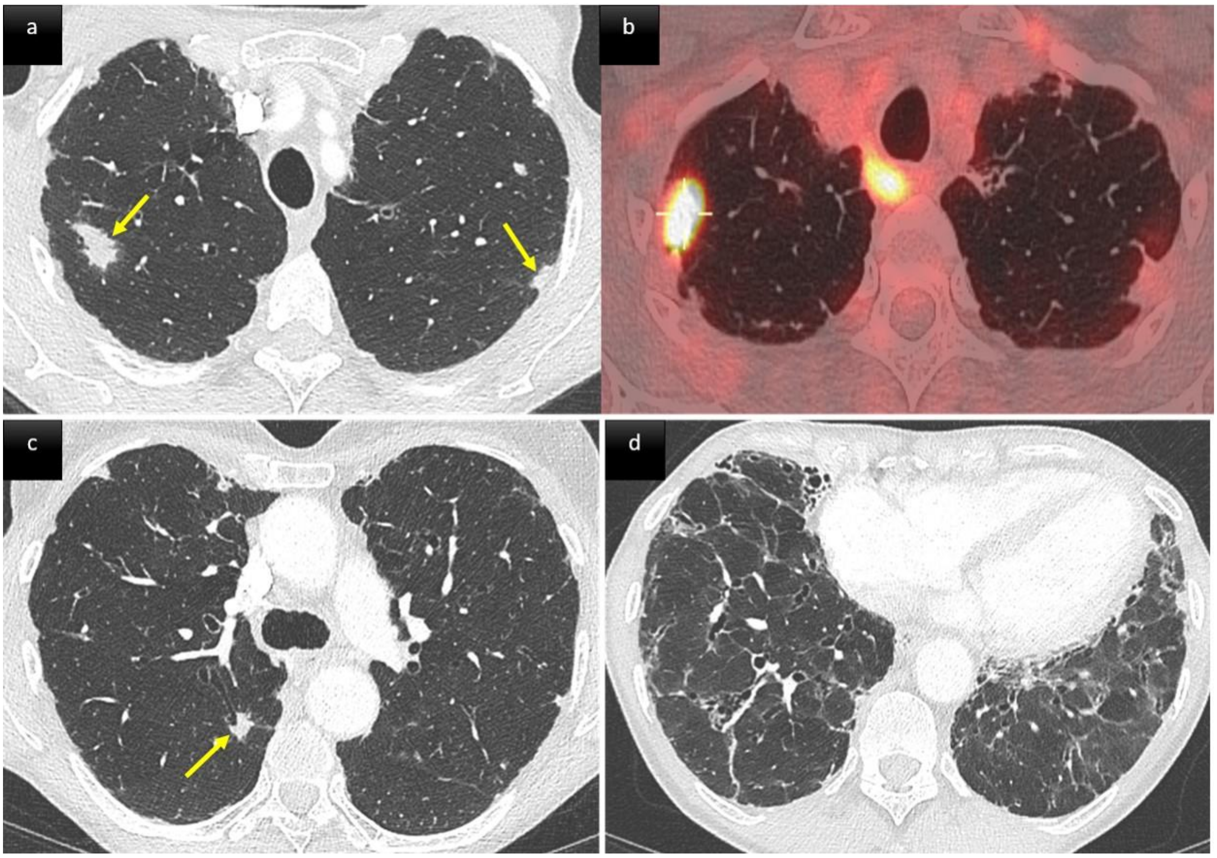

Figure S5: A 71-year-old woman with CVID and GLILD. Unenhanced CT scan (a, c, d) and PET CT scan (b) images. Patchy ground glass and evidence of parenchymal distortion and fibrosis in the lower lobes (d) are regarded as the consequence of longstanding GLILD and repeated infections. The subpleural and broncho centric nodular opacities in the upper lobes (arrows), were slowly progressive over time. The larger nodule showed intense FDG-uptake (b) that led to a CT guided biopsy of the nodule to exclude lymphoma. The histo-pathological result confirmed a new GLILD localization

Figure S6-abcdef_jpeg

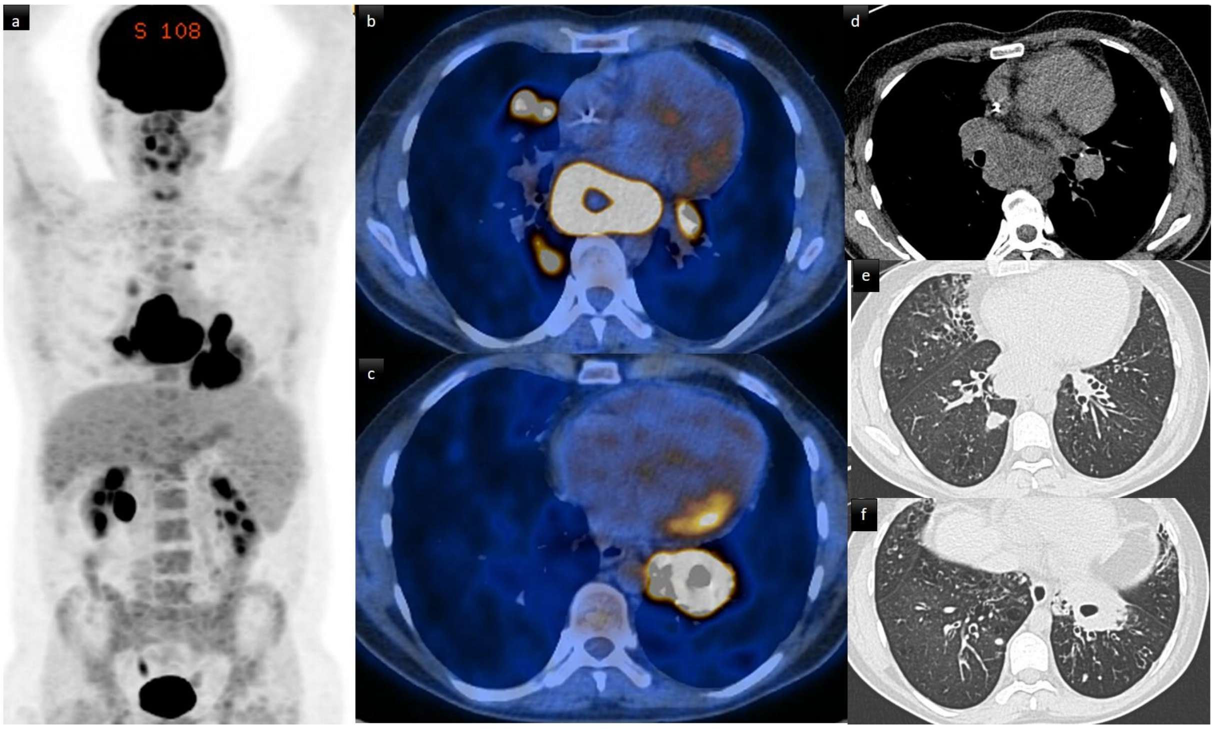

Figure S6: Large B cell non-Hodgkin lymphoma revealing CVID in a 18 yo patient. 18 FDG TEP-CT scan MIP (a) and fusion (b, c) images: large subcarinal necrotic lymphadenopathy, left lower lobe cavitating mass and bilateral lower predominant nodules with intense FDG uptake. Correlation with CT images in mediastinal (d) and parenchymal (e, f) windows. Note the presence of cylindrical bronchiectasis in the middle lobe, lingula and lower lobes, associated with bronchial wall thickening, tree-in-bud and mosaicism, related to recurrent episodes of respiratory tract infections.

Figure S7-abc_jpeg

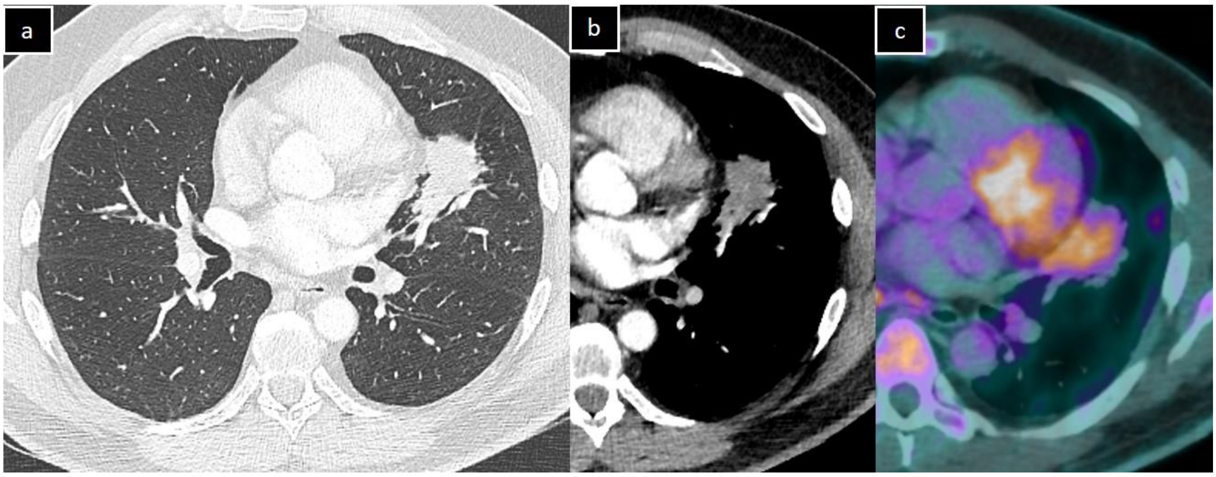

Figure S7- 50 yo male with hyper IgM syndrome. Axial CT images in (a) parenchymal and (b) mediastinal windows show an ill-defined alveolar consolidation in the lingula, with moderate FDG uptake on TEP-CT (c). Bronchoalveolar lavage was normal and the lesion persisted despite 2 courses of antibiotics. Surgical resection provided a diagnosis of MALT lymphoma

Figure S8-ab_jpeg

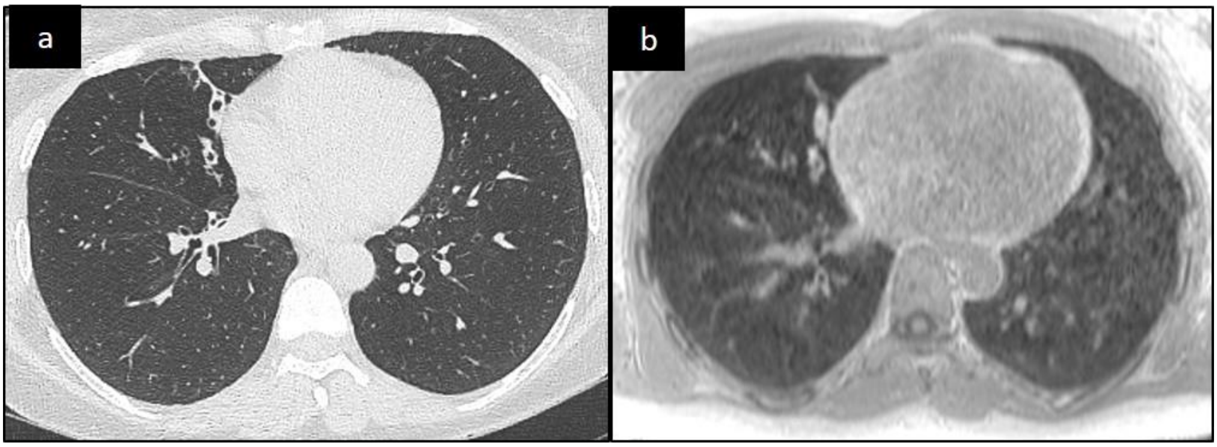

Figure S8- Axial CT (a) and MR (3D UTE) (b) correlation in a 46 yo patient with CVID; cylindrical bronchiectasis and bronchial wall thickening in the right middle lobe

Figure S9-abc_jpeg

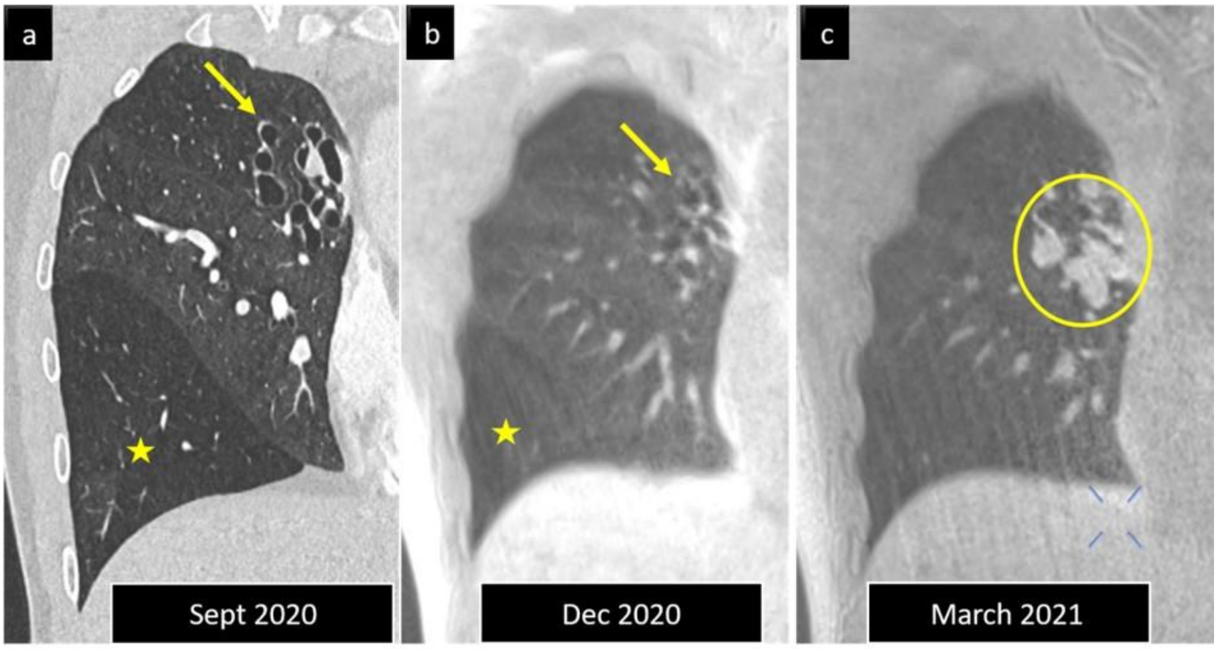

Figure S9- 23 yo male with autosomal dominant HIES; correlation between coronal CT (a) and MR (b, c) scans. To save radiation, the patient is being regularly scanned with MRI and ultra-short TE 3D sequences (b, c). Cylindrical bronchiectasis in the right upper lobe (arrows), and evidence of mosaicism in the right lower lobe (stars). In March 2021, the patient presented with productive cough, fever, and elevated levels of IgE and IgG against *A fumigatus*. Mucus plugging within the airways (circle) consistent with ABPA exacerbation (c).

Figure S10-abc_jpeg

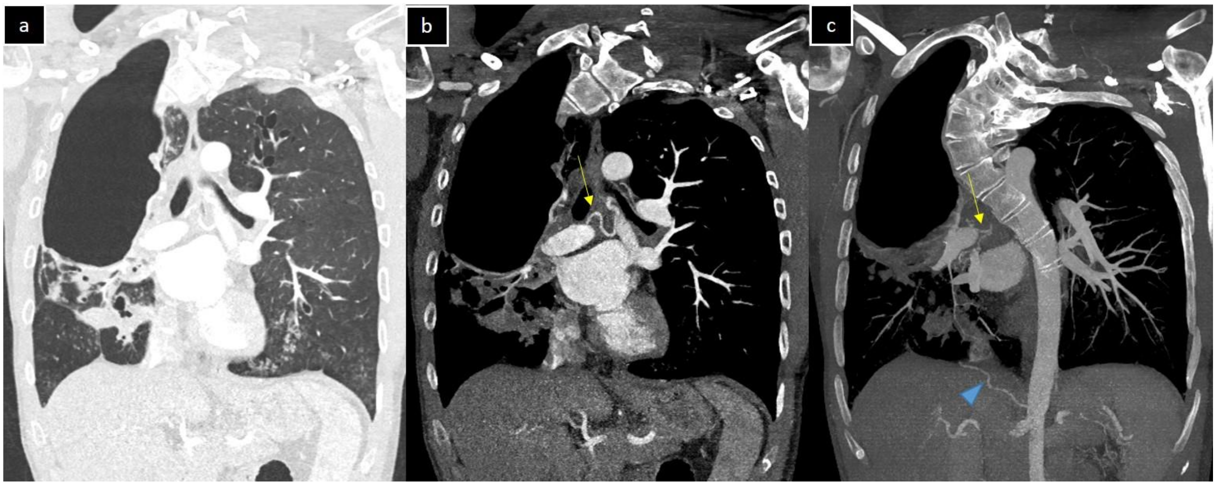

Figure S10- Hemoptysis in a 24 yo patient with autosomal dominant HIES and previous history of chronic cavitating aspergillosis. Coronal post contrast CT images in parenchymal (a) and mediastinal (b) windows. C: coronal oblique image with MIP post treatment. There is a large bulla occupying the right upper lobe (a), associated with enlarged bronchial (arrows) and systemic arteries (arrowhead).

Figure S11-abc_jpeg

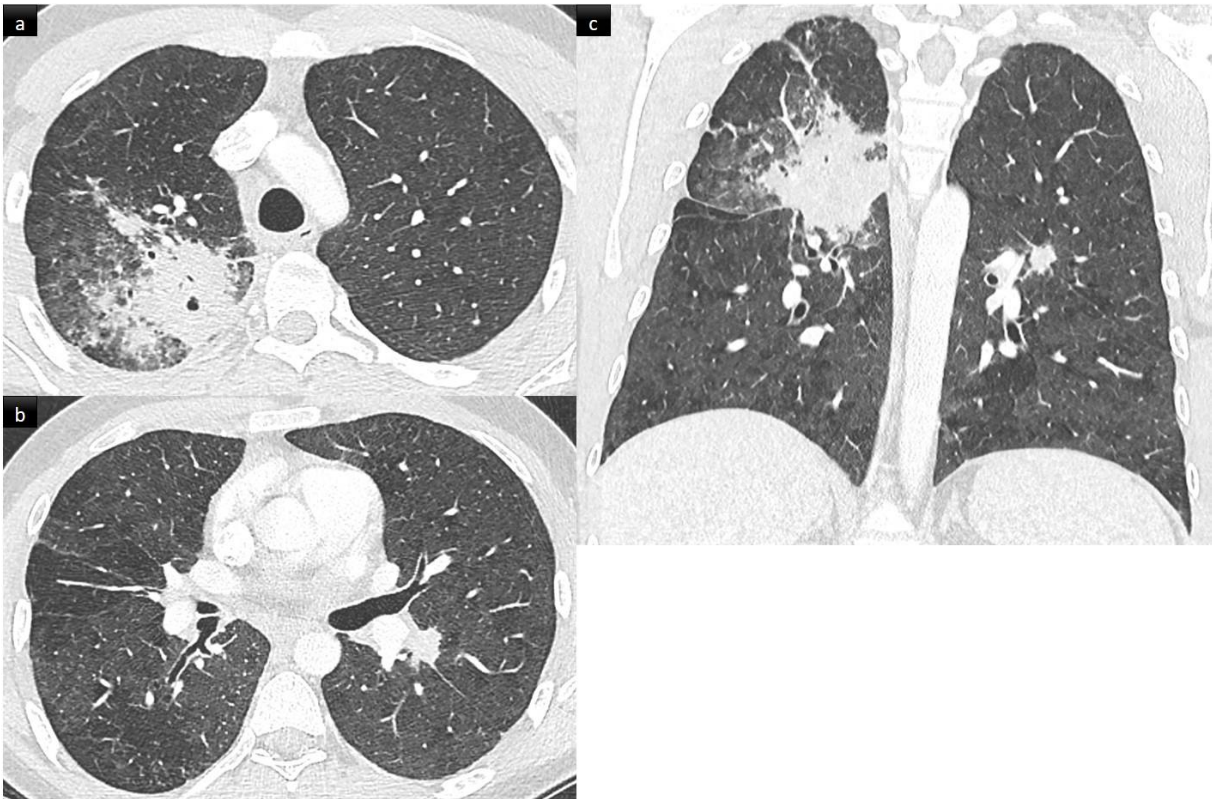

Figure S11: Invasive aspergillosis revealing chronic granulomatous disease in a young patient referred for fever and hemoptysis. Axial (a, b) and coronal (c) CT images in lung window show a large necrotizing mass in the right upper lobe, with transfissural extension towards the right lower lobe. There is a second nodule adjacent to the fissure on the left that reduced with antifungal treatement. Note the rpesence of mosaicism in other parts of the lungs (arrows), consistent with obliterative bronchiolitis and suggesting previous episodes of pulmonary infections.

Figure S12_jpeg

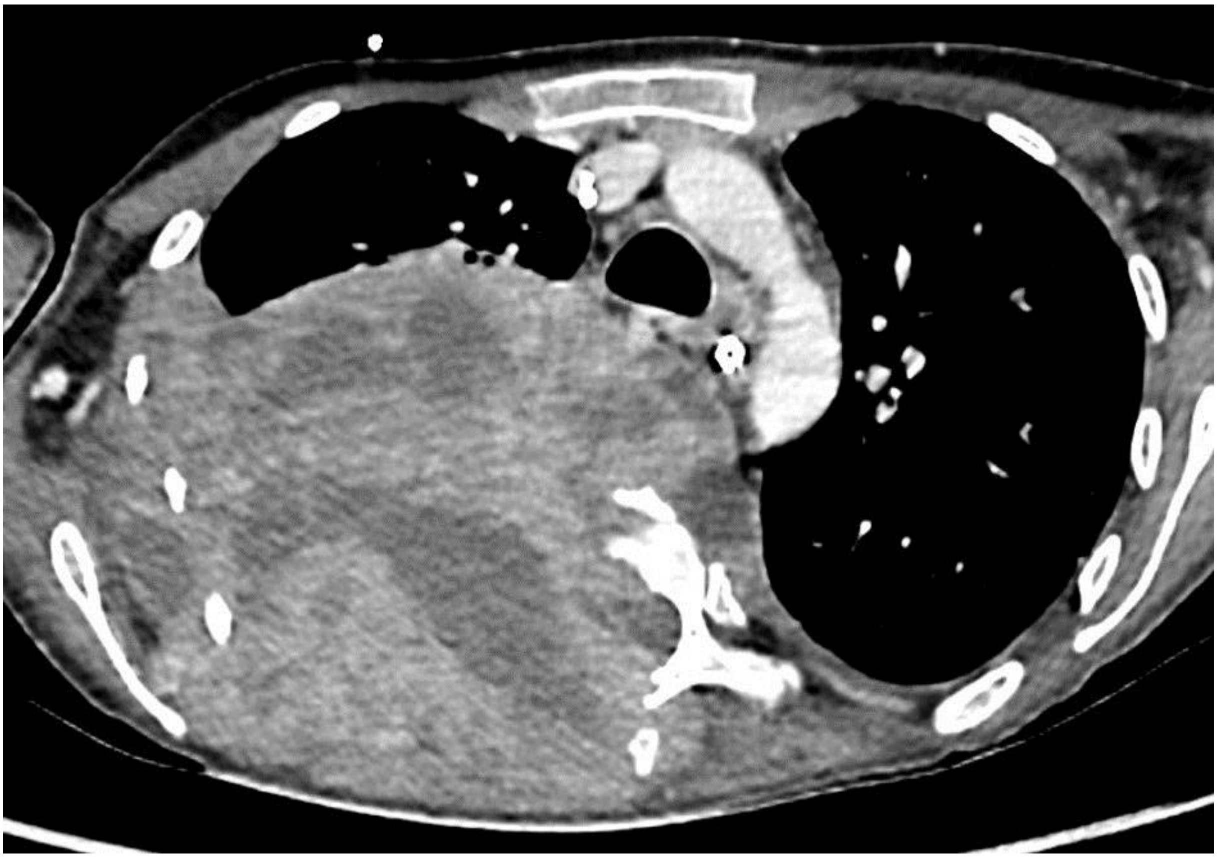

Figuere S-12- Invasive aspergillosis involving the chest wall and the spine in a CGD patient
